# Applying Machine Learning to Predict the Exportome of Bovine and Canine *Babesia* Species That Cause Babesiosis

**DOI:** 10.3390/pathogens10060660

**Published:** 2021-05-27

**Authors:** Stephen J. Goodswen, Paul J. Kennedy, John T. Ellis

**Affiliations:** 1 School of Life Sciences, University of Technology Sydney, 15 Broadway, Ultimo, NSW 2007, Australia; Stephen.Goodswen@uts.edu.au; 2 School of Computer Science, Faculty of Engineering and Information Technology, Australian Artificial Intelligence Institute, University of Technology Sydney, 15 Broadway, Ultimo, NSW 2007, Australia; Paul.Kennedy@uts.edu.au

**Keywords:** *Babesia bovis*, *Babesia bigemina*, *Babesia canis*, *Plasmodium falciparum*, excreted/secreted proteins, exportome, babesiosis

## Abstract

*Babesia* infection of red blood cells can cause a severe disease called babesiosis in susceptible hosts. Bovine babesiosis causes global economic loss to the beef and dairy cattle industries, and canine babesiosis is considered a clinically significant disease. Potential therapeutic targets against bovine and canine babesiosis include members of the exportome, i.e., those proteins exported from the parasite into the host red blood cell. We developed three machine learning-derived methods (two novel and one adapted) to predict for every known *Babesia bovis*, *Babesia bigemina*, and *Babesia canis* protein the probability of being an exportome member. Two well-studied apicomplexan-related species, *Plasmodium falciparum* and *Toxoplasma gondii*, with extensive experimental evidence on their exportome or excreted/secreted proteins were used as important benchmarks for the three methods. Based on 10-fold cross validation and multiple train–validation–test splits of training data, we expect that over 90% of the predicted probabilities accurately provide a secretory or non-secretory indicator. Only laboratory testing can verify that predicted high exportome membership probabilities are creditable exportome indicators. However, the presented methods at least provide those proteins most worthy of laboratory validation and will ultimately save time and money.

## 1. Introduction

*Babesia bovis*, *B. bigemina*, and *B. canis* are tick-transmitted, obligate intracellular, haemoprotozoan parasites of the phylum Apicomplexa [1]. These three species were shown to be the most evolutionarily related following a phylogenetic analysis based on a core set of 975 shared orthologues representing selected *Babesia*, *Theileria* and *Plasmodium* apicomplexan parasites [2]. *Babesia* parasites have the capacity to infect a wide range of vertebrates, including humans, causing a severe disease called babesiosis in susceptible hosts, particularly cattle, horses and dogs [3]. Bovine babesiosis (‘tick fever’) has the potential to occur wherever ticks and cattle have common geographical locations. Consequently, this disease has nearly a worldwide distribution, with most prevalence in subtemperate and tropical areas [3,4]. Annual global economic loss in the beef and dairy cattle industry due to babesiosis is significant and of great concern [5]. No subunit vaccine for bovine babesiosis is widely available [6]. Current vaccines against bovine babesiosis are based on live formulations, which pose a risk of reversion to virulence after administration, with a further possibility of the fully virulent parasite distributing by tick vectors. Subunit vaccines are considered safer, and easier to handle and produce [4]. Canine babesiosis is a clinically significant emerging infectious disease, with a potential worldwide distribution [2,7]. It is currently controlled with varying degrees of success by drug therapy, vaccination with soluble parasite antigens (SPA) from in vitro cultures, and vector control measures [7,8,9]. Several reviews [1,3,5,10,11,12] expand on the current understanding of *Babesia*.

Two biological facts about *Babesia* underpin this study. First, *Babesia* invades mammalian red blood cells (RBCs) and resides freely in the cytoplasm. Mature RBCs do not contain DNA. Consequently, RBCs do not synthesise RNA or possess protein trafficking pathways [13] and simply deliver oxygen to tissues [14]. Second, the structure and function of RBCs are altered following a *Babesia* invasion [15]. Taken together, these two biological facts reinforce current understanding that it is entirely proteins exported from the parasite and not from the host cell that alter structural and functional properties of parasitised RBCs (pRBCs) [10,15]. One alteration is the insertion of parasite molecules at the pRBC surface [16]. These molecules appear to be involved in the attachment of pRBCs to ligands on endothelial cells of blood vessels, a process called sequestration [16,17]. Sequestration is an important contributor to the sequence of events that leads to babesiosis. *B. bovis* pRBCs develop abnormal ridge-like structures on their surfaces and are more rigid than uninfected RBCs [18]. This increased rigidity reduces the overall pRBC deformability, which has been shown to contribute to babesiosis development [19]. In contrast, the ridge-like structures are completely absent on the surface of *B. bigemina* [5] and *B. canis* pRBCs [16]. Both of these species, however, still express parasite antigens at the pRBC surface and cause acute and persistent babesiosis [16,20], although *B. bigemina* has a relatively reduced virulence compared to *B. bovis* [20]. All proteins exported from *Babesia* into the host RBC cytoplasm and/or the RBC membrane (collectively called the exportome) are highly worthy of further investigation. An unknown subset of the exportome is thought to play an important role in altering structural and functional properties of pRBCs mediating the pathogenesis of babesiosis, and such a subset contains potential therapeutic targets [21]. Furthermore, exported proteins exposed to the immune system provide target potential for vaccine development [22].

Malaria parasites (*Plasmodium* spp.) are closely related to *Babesia* in that they are apicomplexans known to infect and modify RBCs [23]. In contrast to babesiosis, malaria is a highly studied disease. A considerable number of proteins are exported during malaria infection; one study documented over 200 from the literature [24] and another [25] predicted 196, although the exact number within the exportome is unknown. A large number of exported *Plasmodium* proteins have been reported to contain an amino acid motif called the *Plasmodium* export element (PEXEL), also known as host-targeting sequence (HT) [26]. The PEXEL motif has been shown to have an association with proteins exported across the parasitophorous vacuole membrane into the RBC cytosol [27]. However, not all exported *Plasmodium* proteins contain the PEXEL motif [14], which highlights the challenge of predicting exported proteins based on a solitary motif. Exported *P. falciparum* proteins can be categorised into two groups based on the presence or absence of the PEXEL motif [28]—a PEXEL motif-containing group, e.g., knob-associated histidine-rich protein (KAHRP) [29]; and a PEXEL-negative exported proteins (PNEPs) group, e.g., erythrocyte membrane protein 1 (PfEMP1) [30]. Only a few *Babesia* proteins have been reported so far that have an association with the RBC membrane [31,32,33], namely, variant erythrocyte surface antigen (VESA) proteins and spherical body protein 2 (SBP2). However, the *Babesia* exportome may be of a similar size to the *Plasmodium* exportome.

*Toxoplasma gondii* is also an apicomplexan pathogen and congenital infections and disease in humans are well known [34]. It is considered the model organism for Apicomplexa [35] and has extensive experimental evidence on their excreted/secreted proteins. *Toxoplasma* is included in the current study as an outlier to *Babesia* and *Plasmodium* because it invades only nucleated cells [36].

The mechanism by which proteins are transported outside haemoprotozoan parasites and inserted into and onto the RBC membranes is still unclear and hotly debated [37,38]. Nonetheless, there are two main protein types that are known to be targeted to the extracellular space: (1) proteins containing an N-terminal signal within the amino acid sequence that targets them via the primary secretion route from eukaryotic cells (the classical or endoplasmic reticulum (ER)/Golgi-dependent secretory pathway) [39]—referred to henceforth as classical proteins, and (2) proteins without an N-terminal signal that are exported by distinct non-classical secretion pathways [40]—referred to henceforth as non-classical proteins.

Many programs have been developed to predict protein subcellular locations in eukaryotes (http://bioinformatics.ysu.edu/tools/subcell.html [Date last accessed: 27 May 2021]), but not specifically for exportome prediction. Most prediction programs are amino acid sequence-based, focusing on either delivery signals/motifs within the protein amino acid sequence [41] or amino acid composition. The best known delivery signal is the secretory signal peptide (SP). Although there are no simple SP consensus sequences, three distinct compositional regions on the amino acid sequence help define SPs: N-terminal, central hydrophobic, and C-terminal regions. Specific motifs targeting the protein to specific subcellular compartments are within the N-terminal, and the signal peptidase cleavage site preceding the mature protein is within the C-terminal region. Signal peptides are cleaved off while the protein is translocated through the parasite membrane [42]. It is important to highlight that not all exported proteins have SPs and not all SP-containing proteins are secreted beyond the parasite membrane [43]. Furthermore, there are two distinct types of SP-containing proteins associated with RBCs: proteins involved in RBC invasion [44], e.g., merozoite surface antigen-1 (MSA-1) [45], thrombospondin-related anonymous protein (TRAP) [46], and rhoptry associated protein 1 (RAP-1) [47,48]; and exported proteins involved in RBC modification, e.g., VESA and SBP2.

An alternative to predicting SPs is grounded on the observation that proteins delivered to distinct subcellular compartments (e.g., cytoplasmic, extracellular, mitochondrial, nuclear) have different amino acid compositions [49]. Several programs such as SecretomeP [50] and OutCyte [51] specifically predict non-classical proteins.

The genome sequence of the virulent Texas T2Bo isolate of *B. bovis* was made publicly available in 2007 [6]. Gohil and colleagues [21] were the first to predict a T2Bo exportome using a rule-based bioinformatics approach. The rules were based on known characteristics of *Plasmodium* exportome proteins such as presence of SP but no transmembrane (TM) domain(s) and glycosylphosphatidylinositol (GPI) anchors. The Wellcome Sanger Institute sequenced the genome of an established virulent Australian BOND isolate of *B. bigemina* in 2013 and updated it in 2017; and the genome sequencing of a virulent *B. canis* Hungarian field isolate (strain BcH-CHIPZ) was led by the Institute of Parasitology, University of Zurich in 2017 [2].

We present three machine learning (ML) methods to predict exportome membership probabilities for every known *B. bovis* T2BO, *B. bigemina* BOND and *B. canis* BcH-CHIPZ proteins. The methods use both delivery signals and amino acid composition obtained from a collection of existing subcellular prediction programs and novel (or adapted) amino acid composition algorithms. Collected data are converted for input to an ensemble of ML algorithms trained on classical proteins. SecretomeP and OutCyte are used to predict exportome membership probabilities for non-classical proteins. The classical- and non-classical-derived probabilities provide an informed starting point for laboratory testing, i.e., the desired percentage of top-ranked exportome proteins can be selected to suit laboratory capability and budget.

## 2. Results

### 2.1. Rule-Based Approach for Predicting an Exportome

The Gohil study [21] provides the only known list of predicted *B. bovis* exportome proteins, and consequently the method used to predict these proteins represents the current state-of-the-art prediction method for exportome membership. The method used was a rule-based approach applied to predicted protein characteristics. That is, a protein was classified an exportome protein based on the rule it contained an SP but no TM domain and no GPI anchors. In the Gohil study, SPs were predicted by SignalP 3.0 [52], TMs by TMHMM 2.0 [53], and GPIs by DGPI (which is no longer available online)—214 proteins were reported as belonging to the *B. bovis* T2Bo exportome, and 3286 proteins as ‘non-secretory’. The 214 published exportome proteins were used as the ‘positives’ training dataset in this current study. Using this dataset was deemed the best option given that there is currently no laboratory verified list of exportome proteins for *B. bovis*. A ‘negatives’ training dataset was created by randomly selecting 214 from the 3286 non-secretory proteins using the Python random module.

A new version of SignalP (version 5.0) has been released since the Gohil Study. In the current study, the latest SignalP, the current TMHMM (version 2.0) and PredGPI (version 1.0) [54] were used to predict the required rule-based characteristics for the 214 Gohil exportome proteins. The predicted characteristics are shown in Supplementary Materials Table S1 (an Excel file). A SignalP score greater than 0.5 is a SP presence indicator. In summary, the latest SignalP predicts 35 of 214 exportome proteins to have no SP.

### 2.2. Amino Acid Frequency

The frequency for each of the 20 amino acids within the training dataset sequences was counted. The counts for the entire protein length are reported along with two graphs in Supplementary Table S1 on sheet ‘Entire’. One graph uses the counts directly and the other has weighted counts with respect to protein length. The weighted graph shows no detectable distinguishing differences between the positives and negatives. In Supplementary Table S1 on sheet ‘Start_Centre_End’, three graphs show the total counts for the first 25, end 25, and the remaining centre amino acids, respectively. Distinguishing differences can be observed for the first and end regions.

Graphs on sheets ‘Dipeptide_entire’, ‘Dipeptide_start’, and ‘Dipeptide_end’ show counts for the frequency of two consecutive amino acid combinations for the entire length, first 25, and end 25 of the training sequences (i.e., dipeptide counts, e.g., AA, AC, AD—400 possible combinations). The graph for the entire length has non-distinguishable differences, whereas the first 25 graph clearly highlights differences between positives and negatives. The end 25 graph still shows differences but not as prominent as for the start.

Tripeptide, e.g., AAA, AAC (8000 combinations) and quad-peptide, e.g., AAAA, AAAC (160,000 combinations) frequencies were also counted for the first and end 25 amino acids. The counts are shown in Supplementary Table S1 on sheets tripeptide_start, tripeptide_end, quad_peptide_start, quad_peptide_end. These counts show that certain combinations of amino acids such as ‘VVA’ and ‘VAFG’ occur too frequently in the positives in comparison to the negatives to be a random phenomenon.

Supplementary Table S2 analyses the amino acid counts at each specific position in the first and end 25 amino acids in the positives and negative datasets. Clear differences can be observed between the positives and negatives, particularly at the start. For example, at position #2 from the start of the positive protein sequences, valine (V) is the most frequent, with a count of 43 out of 214 (20.09%) and an expected count of 14 (6.64%) based on the frequency of valine in all 3706 known *B. Bovis* T2Bo sequences. Therefore, the occurrence of V is more than twice the expected.

### 2.3. Machine Learning Approach for Predicting an Exportome

**Figure 1:**
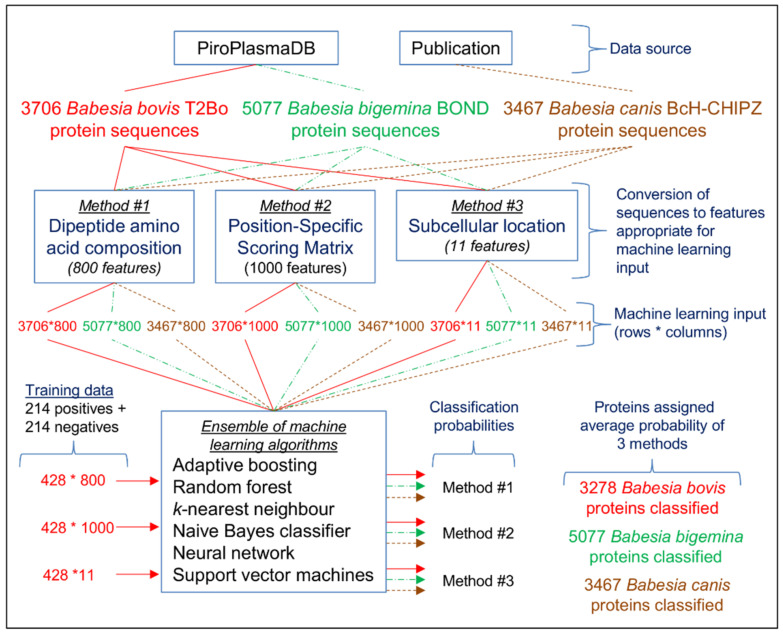
This schematic shows the steps taken to predict *Babesia bovis* T2Bo, *Babesia bigemina* BOND, and *Babesia canis* BcH-CHIPZ exportomes. Three independent methods (dipeptide amino acid composition, position-specific scoring matrix, and subcellular location) are used to convert protein sequences from the PiroPlasmaDB database and Publication (Eichenberger et al., 2017) into formats appropriate for machine learning (ML) input (features are what are used by ML algorithms to help make predictions). The data flow is highlighted in red for *Babesia bovis*, green for *B. bigemina*, and brown for *B. canis*. Training data for ML are taken from *B. bovis*, where positives are proteins expected to be exportome members and negatives are non-secretory proteins. The number of classified *B. bovis* proteins is 3278 (i.e., 3706 total minus 428 training).

Three independent methods (dipeptide amino acid composition, position-specific scoring matrix, and subcellular location) were applied to make consensus predictions for *B. bovis* T2Bo, *B. bigemina* BOND, and *B. canis* BcH-CHIPZ exportomes (Figure 1). An ensemble of ML algorithms was used to classify proteins as exportome or non-exportome based on a classification probability. The ML input training file for each method consisted of *B. bovis* protein sequences representing 214 classical exportome (i.e., positives) and 214 non-exportome (i.e., negatives) members as published in the Gohil paper.

### 2.4. Method #1—Dipeptide Amino Acid Composition

**Figure 2:**
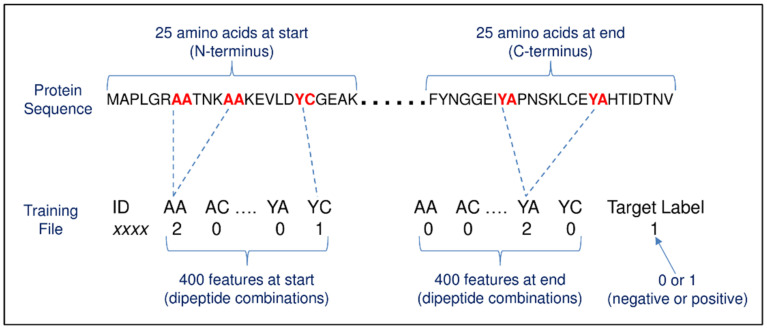
This schematic shows the conversion of protein sequences to fixed length features for machine learning input using dipeptide amino acid composition Protein sequences are typically of varying length. Machine learning (ML) requires a uniform set of features. Here, each feature is the frequency of a dipeptide within a sequence—400 possible dipeptide combinations (i.e., 20 amino acids raised to the power of two consecutive amino acids). The protein sequence for ML training is therefore represented by 800 features (400 at the start and 400 at the end). The target label is what the ML algorithm attempts to predict, i.e., exportome (positive) or non-exportome (negative), and features are what the ML algorithm uses to help make the prediction.

Method #1 was adapted from a previous study [24] that predicted secretory proteins of malaria parasites based on dipeptide amino acid composition using SVM. The current study uses 800 features per protein representing dipeptide amino acid counts of 400 dipeptide combinations within the first 25 amino acids at the start (N-terminus) and 400 within the last 25 amino acids at the end (C-terminus) of the training sequences (Figure 2). The results from 10-fold cross validation are shown in Table 1 and extended in Supplementary Table S3 on sheet ‘Evaluation of algorithms’. In summary, when using all six evaluated algorithms in the ensemble—adaptive boosting (AdaBoost), support vector machines (SVM), random forest (RF), artificial neural network (ANN), *k*-nearest neighbour classifier (*k*NN) and naive Bayes classifier (NB)—the accuracy was 89.72%, with the poorest performances from *k*NN and NB. The best accuracy (90.89%) was achieved with an ensemble of adaBoost, SVM, RF, ANN. Classification probabilities are listed in Supplementary Table S3 on sheets ‘Positives’ and ‘Negatives’ for this ensemble. The accuracy was reduced to 90.65% when using the same ensemble but only with the first 25 amino acids (400 features per protein), i.e., the protein end sequence contributes a small fraction to the overall performance but is still worth the inclusion. When the features were tripeptide counts from the first 25 amino acids (8000 features per protein), the best accuracy (87.38%) was achieved by SVM. To further evaluate the best performing model as determined by cross validation, the training data were randomly split into three parts: 70% used for training, 15% for validation (with parameter tuning), and 15% for testing. Each part had an equal number of exportome and non-exportome proteins. Random data splitting followed by measuring the model’s performance on the test data was repeated 100 times and the measures averaged. The best performing algorithm was SVM with an 88.58% average accuracy, whilst the ensemble had an accuracy of 88.34% in comparison to the cross validation-derived 90.89% (details are shown in Supplementary Table S3 on sheet ‘Test results’).

Ten-fold cross validation was also performed on reduced numbers of features to assess its impact on overall performance. Large numbers of features compared to small numbers of training examples may lead to overtraining. A variable importance table generated by the randomForest R package that ranks the importance of each feature was used as an aid for feature selection. The ensemble accuracy using all features (i.e., 800) was 90.89% in comparison to 90.19%, 89.72%, and 91.12% for 300, 200, and 100 features, respectively. Only marginal differences were observed for individual algorithms when comparing performances between reduced feature numbers and all features. SVM was the exception, with an average reduced accuracy of 33% (results expanded in Supplementary Table S3 on sheets ‘Importance’ and ‘Importance evaluation’).

### 2.5. Method #2—Position-Specific Scoring Matrix (PSSM)

**Figure 3:**
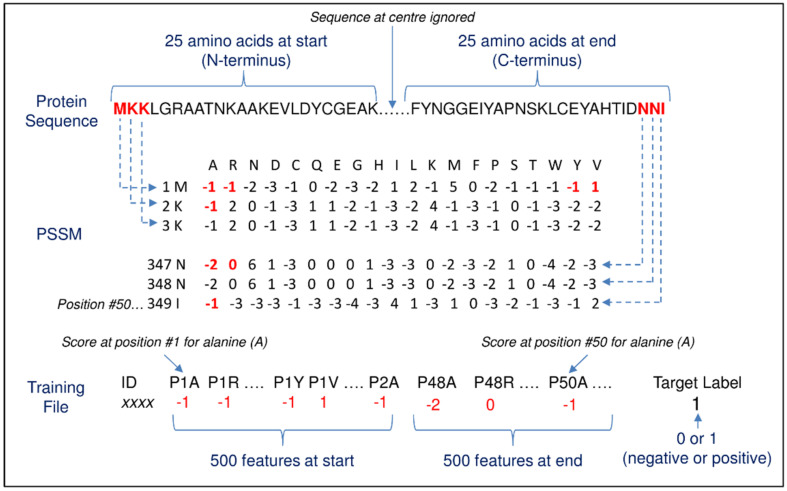
This schematic shows the conversion of protein sequences to fixed length features for machine learning using position-specific scoring matrix (PSSM). Here, a protein sequence is first converted to a position-specific scoring matrix (PSSM). In the PSSM, there are 20 columns for each genetic code amino acid and one row for each amino acid in the primary protein sequence. Values in the matrix are a normalised frequency count of each genetic code amino acid at the same position in a protein multiple sequence alignment, e.g., the scores are A = −1, R = −1, etc. for position #1 containing the sequence M (features P1A and P1R). Only PSSM scores for the 25 amino acids at the sequence start and end are used as features—1000 feature scores in total (i.e., 2 * 20 genetic code acids * 25 sequence positions).

Method #2 was also inspired by the Verma study [24], which used position-specific scoring matrix (PSSMs) [55] to predict malarial secretory proteins. PSSM is a common representation of motifs in biological sequences as an alternative to consensus sequences. In the current study, matrix values for the first and last 25 amino acids of the training sequences were extracted from PSSMs to form 1000 features per protein (Figure 3). Table 2 shows the results from 10-fold cross validation.

In summary, RF had the best accuracy with 89.95%. The overall performances were on par with the dipeptide amino acid composition method. Refer to Supplementary Table S4 on sheet ‘Evaluation of algorithms’ for expanded results and on sheets ‘Positives_methodology#2’ and ‘Negatives_methodology#2’ for classification probabilities obtained from adaBoost, SVM, RF, ANN, and the ensemble. The ensemble average accuracy on test data from a train (70%)–validate (15%)–test (15%) split repeated 100 times was 92.09%, although RF had the best accuracy with 92.81% (details in Supplementary Table S4 on sheet ‘Test results’). Following 10-fold cross validation with reduced feature numbers, the ensemble accuracy when using all features (i.e., 1000) was 89.95% in comparison to 91.19%, 91.43%, and 91.90% for 300, 200, and 100 features, respectively. Similarly to Method #1, only SVM had a significant reduction (26%) in accuracy (results expanded in Supplementary Table S4 on sheets ‘Importance’ and ‘Importance evaluation’).

### 2.6. Method #3—Subcellular Location

Method #3 used six freely available programs (SignalP [56], WoLF PSORT [41], TargetP [43], TMHMM [53], Phobius [57], and DeepLoc [58]) to predict various protein characteristics encoded within the training sequences that are related to subcellular localization. Eleven prediction features per protein were extracted from the six program outputs to form the ML input. Table 3 shows the results from 10-fold cross validation. In summary, an ensemble of adBoost, ANN, kNN, NB; RF, SVM had the best accuracy with 96.26%. Supplementary Table S5 on sheets ‘Positives’ and ‘Negatives’ show the classification probabilities obtained from the ensemble along with individual scores from the six programs. The ensemble average accuracy on test data from a train (70%)–validate (15%)–test (15%) split repeated 100 times was 95.94% (details in Supplementary Table S5 on sheet ‘Test results’).

### 2.7. Comparison of Classification Outcomes from the Three Methods

Supplementary Table S6 on sheet ‘FP_FN_comparison’ shows comparisons of the false-positive and -negative outcomes from the ensemble of the three methods. It highlights proteins consistently misclassified: one conserved hypothetical protein (consistently a false positive) and five proteins consistently false negatives—mac/perforin domain-containing protein, translation elongation factor G (EF-G) putative, and three hypothetical proteins.

### 2.8. Full Babesia Bovis T2Bo Exportome Prediction

The training data were adjusted accordingly based on classification probabilities of proteins highlighted to be consistently misclassified, i.e., two proteins swapped between the negative and positive datasets—conserved hypothetical proteins (BBOV_IV000860 and BBOV_II004000); and four proteins removed from the negative dataset—mac/perforin domain-containing protein (BBOV_II002020), translation elongation factor G (EF-G) putative (BBOV_IV004710), and hypothetical proteins (BBOV_IV004200 and BBOV_IV008420). Adjusting training datasets is normally not recommended as it would lead to overfitting, especially when it consists of verified examples. In this study, however, the adjusted proteins are deemed to have had incorrect classifications in the cross validation training data owing to the fact their initial classifications were unverified in the Gohil study. All *B. bovis* proteins (minus the adjusted training data) were classified using the ensemble from the three methods. Supplementary Table S7 lists the classification probabilities of the three methods plus an average of these probabilities—144 proteins out of 3282 have an average probability greater than 70%; 89 greater than 80%, and 40 greater than 90% of being correctly classified in the exportome class.

The reliability of annotated protein names for *Babesia* species is considered poor by the current study (see later Discussion). Despite this, highest-scoring proteins are assessed here on their names. The highest-scoring protein is one of the 44 small open reading frame (smORF) proteins [59]. Another high-scoring named protein is SBP2. Based on current knowledge, smORF and SBP2 are considered here as worthy exportome members. SBP2 proteins are shown to be released from spherical bodies following invasion and are believed to be responsible for RBC modifications [31,60,61]. SmORF proteins are known exported proteins that are proposed to play a role in variant erythrocyte surface antigen (VESA) protein biology [6], i.e., smORFs could assist in sequestration together with VESA1 proteins. Interestingly, smORFs have not been detected in *B. bigemina*, suggesting that they are involved, either directly or indirectly, in the development of the ridge-like structures on pRBC surfaces [20].

VESA proteins are the main known *B. bovis* RBC surface-exposed proteins. Only three out of 123 VESA proteins had an exportome probability greater than 50%. This result was not unexpected because VESA proteins contain no N-terminal signal sequence and a TM domain.

Of the 144 proteins with an exportome probability greater than 70%, two have smORF in their name, 4 have SBP2, one erythrocyte membrane-associated antigen, 62 hypothetical, 34 membrane protein (putative), and 42 with non-generic names. The named proteins other than SBP2, smORF, and ‘erythrocyte membrane-associated antigen’ are not known be exported. One notable high-scoring named protein is MAC/perforin domain-containing protein, which may be a possible exportome member. This protein has been shown to be secreted from micronemes during the intraerythrocytic parasite stage and bind to the pRBC membrane, where it facilitates the egress of the parasite [62]. A high-scoring protein named ‘membrane, putative’ (BBOV_III004280) has been experimentally verified in a recent study [60] to be an exported protein and a novel virulence factor for *B. bovis*.

### 2.9. Full Babesia Bigemina BOND Exportome Prediction

All *B. bigemina* BOND proteins were classified using the three methods with the *B. bovis* training data. Supplementary Table S8 lists the classification probabilities of the three methods and is ordered on average probability—371 proteins have a probability greater than 70% and are considered here as the *B. bigemina* exportome. Most (96%) of the 371 proteins contain hypothetical or putative in their name. High-scoring known exported proteins were SBP3 and SBP4. Notable named proteins with possible exportome membership were MAC/perforin domain-containing proteins.

### 2.10. Full Babesia Canis BcH-CHIPZ Exportome Prediction

All *B. canis* BcH-CHIPZ proteins were classified using the three methods with the *B. bovis* training data. Supplementary Table S9 lists the classification probabilities of the three methods and is ordered on average probability—196 proteins have a probability greater than 70% and are considered here as the *B. canis* exportome. The majority (59%) of names for the 196 contain hypothetical or unnamed. There are no names associated with known exported proteins except possibly MAC/perforin domain-containing protein.

The column ‘Predicted route of export’ contains the predicted export route as obtained from the *B. canis* genome sequencing study [2]. Proteins with the route ‘cSP-containing’ (signal peptide containing) are considered to be the most likely members of the *B. canis* exportome in this current study. There are 148 ‘cSP-containing’ proteins for which 122 (82%) were predicted by the combined three methods to be exportome members.

### 2.11. Comparison between Babesia Exportome Predictions

The highest-scoring exportome proteins from each of the three species were compared to provide insights into babesiosis pathogenicity. In Supplementary Table S7, sheets ‘T2Bo_BOND_comparison’ and ‘T2Bo_ BcH-CHIPZ_comparison’ show the results from a BLASTP comparison between the 358 *B. bovis* exportome proteins (144 novel + 214 from training data) with all *B. bigemina* and *B. canis* proteins. The important columns are ‘Exportome’ (a YES or NO if the matching *B. bigemina* or *B. canis* protein was also predicted to be an exportome protein, i.e., >0.7 average for matching protein (a 0.7 rather than a 0.5 threshold was applied to have potentially less false positives to be tested in the laboratory); and ‘Comparison_status’ (<25% or 25–75% or >75% sequence similarity but taking into account query coverage). Here, *B. bovis* proteins <25% are considered unique exportome proteins and >75% as common exportome proteins. The assumption is that the unique proteins are more likely contributing to the formation of the ridges than the common exportome proteins. Proteins such as SBP2, smORF, and MSA-1 were identified as unique.

Similarly in Supplementary Table S8, sheet ‘BOND_T2Bo_comparison’ and ‘BOND_BcH-CHIPZ _comparison’ contain a BLASTP comparison between the 371 predicted *B. bigemina* exportome proteins with all *B. bovis* and *B. canis* proteins. The most notable result is that all the unique exportome proteins when compared to *B. bovis* are hypothetical proteins, which suggests their functions are still to be determined. In Supplementary Table S9, sheet ‘BcH-CHIPZ_T2Bo_comparison’ and ‘BcH-CHIPZ_BOND_comparison’ contain a BLASTP comparison between the 207 predicted *B. canis* exportome proteins with all *B. bovis* and *B. bigemina* proteins. Bc28 protein family (28 × 2) is one of the unique proteins when compared to *B. bovis* but has a 25.2% similarity to a hypothetical *B. bigemina* protein; and BcMSA1 is a unique protein when compared to *B. bigemina*.

Some named proteins that are common to all three species are heat shock proteins (HSP70 and HSP90). HSP70 has been shown to be regulated by DnaJ proteins [61]. Exported DnaJ proteins are thought to have several possible roles including remodelling of membrane compartments in the RBC cytoplasm, and in the assembly of correctly folded knobs and transporters at the RBC membrane [61]. Two DnaJ domain-containing proteins (BBBOND_0306800 and BBOV_I000970) are high-scoring exportome members.

### 2.12. Non-Classical Exported Proteins

SecretomeP [50] and OutCyte [51] were used to predict the probability of being exported by a non-classical secretory pathway for every *B. bovis*, *B. bigemina*, *B. canis* protein. Supplementary Table S10 contains the results. OutCyte and SecretomeP have many contradictory predictions. For example, OutCyte predicted 627 non-classical proteins that were not predicted by SecretomeP, and conversely, SecretomeP predicted 749 non-classical proteins that were not predicted by OutCyte. Therefore, a protein was only classified ‘non-classical’ when both OutCyte and SecretomeP predicted it as non-classical, i.e., an average of the OutCyte and SecretomeP non-classical probability scores was determined, and proteins with an average score greater than 0.7 are considered here to be exported proteins worthy of further investigation. In summary, 409 *B. bovis* proteins were predicted as non-classical (although 7 of these proteins are also predicted as classical), 465 *B. bigemina* proteins were predicted as non-classical (4 classical), and 574 (8 classical) for *B. canis*.

### 2.13. Plasmodium Falciparum and Toxoplasma Gondii Exportome Prediction

The three exportome prediction methods were applied to all *Plasmodium falciparum* 3D7 and *Toxoplasma gondii* ME49 proteins. *Plasmodium* represents a major group of RBC parasites in contrast to the outlier *Toxoplasma* which does not live in RBCs. Furthermore, *P. falciparum* and *T. gondii* are well-studied species, and 3D7 and ME49 are their respective reference genomes. Supplementary Table S11 lists the classification probabilities of the three methods in addition to PEXL motif, motif location, SP location, and non-classical scores. Many exported *Plasmodium* proteins have an SP followed by a PEXEL motif.

There is no known list of experimentally validated exportome proteins for *B. bovis*, *B. bigemina*, and *B. canis*. However, several exported proteins for *P. falciparum* have been reported in multiple publications, namely, PfEMP1 [30], DnaJ protein [61], GBP130 protein [29], KAHRP [29], ring-infected erythrocyte surface antigen (RESA) [63], *Plasmodium* helical interspersed subtelomeric (PHIST) [25], serine/threonine protein kinase [64], repetitive interspersed family (RIFIN) [65], and subtelomeric variable open reading frame family (STEVOR) [29]. PlasmodDB lists 196 *P. falciparum* proteins predicted to be exported by ExportPred [25] (186 PEXEL-motif-containing and 10 PNEPs). The list includes proteins reported to be exported or have an association with RBCs. The 196 proteins formed the *Plasmodium* benchmark dataset.

Two *Toxoplasma* subcellular organelles, rhoptries (ROPs) and dense granules (GRAs) are known to excrete-secrete their proteins into the parasitophorous vacuole and/or host cell [66]. For the *Toxoplasma* benchmark dataset, 19 GRA and 19 ROP proteins were used. The 196 *P. falciparum* and 38 *T. gondii* proteins representing possible exported or excreted/secreted proteins were used to assess whether the *B. bovis* training data generalised to *Plasmodium* and *Toxoplasma*, i.e., whether signals encoded in *Babesia* amino acid sequences that distinguish exported proteins are similar in related parasites. For the 196 *P. falciparum* proteins, 186 (95%) contain a PEXEL motif; and 105 (54%) have a SP predicted by SignalP 3.0 but only 61 (31%) by SignalP 5.0. There were only 59 (30%) with a PEXEL motif and SP, where 50 were either RIFIN or STEVOR proteins that are thought to play roles in export and display of virulence proteins [14,61]. The motif location for the 59 proteins was 13–27 amino acids downstream of the SP (SP cleavage sites and PEXEL motifs were located 17–28 and 35–51, respectively, in contrast to motif locations 9-1832 when considering all 196 proteins).

No *P. falciparum* 3D7 proteins were predicted as non-classical when using a consensus of OutCyte and SecretomeP. However, OutCyte predicted 23 non-classical proteins and all were *P. falciparum* PfEMP1. There are 25 PfEMP1 proteins in the 196 benchmark dataset. PfEMP1 is a known non-classical protein with no SP but importantly plays a role in RBC modification [30]. It acts as a major virulence protein anchored on knob-like structures at the RBC membrane mediating adhesion to endothelial cells [61,67]. PEXEL motifs were found in all 25 PfEMP1 proteins but located in the range 128–1832 from the N terminal.

The three methods classified 60 of the 196 (30%) as exportome members when using a 0.5 threshold applied to the average score. This low percentage was not unexpected as the *B. bovis* training data only comprise proteins with SPs and no TMs. Method #1, however, successfully classified 59 (100%) out of the 59 with a PEXEL motif and SP (whereas Methods #2 and #3 classified only 17%). The low percentage for Method #2 suggests that PSSMs representing *Babesia* exported proteins in the training data do not adequately generalise to *Plasmodium*. The positive training data for Method #3 purposefully contain proteins with no TM domains. Method #3 therefore has a low classification because 55 of the 59 contain TM domains. Method #1 counts dipeptides in the first 25 amino acids. An N-terminal SP is typically 15–25 amino acids in length with the main sorting signal thought to be at the start. The perfect result suggests that the dipeptide counts that distinguish between exported and non-exported proteins are associated with the SP, and this distinguishing pattern in the *Babesia* training data generalises to *Plasmodium*. Furthermore, the PEXEL motif is obviously not even taken into account by Method #1 because the motif is typically downstream of the first 25 amino acids. A further 58 *P. falciparum* proteins were classified here as possible exportome members based on the presence of a PEXEL motif located less than 51 amino acids downstream of a SP cleavage site and having a Method #1 probability greater than 0.7. The 58 exportome members included 37 RIFIN, six STEVOR, and five named ‘*Plasmodium* exported protein’.

For the 38 *T. gondii* proteins, 14 (37%) contain a PEXEL motif of which 8 have a SP predicted by SignalP. The low PEXEL percentage and wide range of motif locations (57-1001) suggests that PEXEL is not an indicator of excreted/secreted *T. gondii* GRA and ROP proteins. Note that a PEXEL-like motif specific to *Toxoplasma* has been proposed as a signal for protein cleavage but is not an indicator of export into the host cell [68]. SignalP 5.0 predicted 23 (61%) to have a SP. The SP cleavage site range was noticeably broader (18–48) than for *Plasmodium* and 16 of the 23 had a site location greater than 25. The three methods classified 23 (61%) as exportome members when using a 0.5 threshold applied to the average score. Predictions from each individual method were about the same (63, 58 and 55% for Methods #1, #2 and #3, respectively). Method #1 performed poorly possibly because the SP or SP regions were beyond Method #1’s ‘25 amino acids range’ for most of the *T. gondii* GRA and ROP proteins. The results from all three methods suggest that *Babesia* training data are unsuitable for *Toxoplasma*.

The location of the first PEXEL motif when searching from N to C terminals was determined for all *B. bovis* proteins. At least one PEXEL motif was found in 35% of the proteins, which is a similar result (27%) with all *P. falciparum* proteins. When considering only the 337 *B. bovis* proteins predicted here to be exportome members, 84 (25%) have a PEXEL motif. However, the location for 78 of the 84 is beyond 51 (*Plasmodium* PEXEL motifs are mostly located 35–51). These results imply that a PEXEL plays an inconsequential part, if any, in the export of *Babesia* proteins.

## 3. Discussion

The goal of the current study was to predict from protein sequences, the exportome members for three closely related *Babesia* species, *B. bovis*, *B. bigemina*, and *B. canis*. An exportome member here is a protein that is transported outside the parasite and then inserted into the host RBC cytoplasm and/or the RBC membrane. These exported proteins are considered worthy candidates as drug and/or vaccine targets. One of many challenges to this endeavour is that we do not yet know what constitutes a complete exportome to verify predictions. Furthermore, the types of proteins that may represent a complete exportome are also unknown. What we do know, nonetheless, is that primary protein sequences contain signals that govern transport and localization of newly synthesised proteins. With this in mind, our expectation was that exportome proteins would have different delivery signals and/or amino acid composition to those proteins not associated with the RBC membrane. We included data from *P. falciparum* and *T. gondii* in the study design, as they provide important benchmarks for this study as extensive experimental evidence is available on their exportome or excreted/secreted proteins.

The Gohil study currently provides the only list of *B. bovis* T2Bo proteins (214 in total) expected to be exportome related. Only five of these proteins were tested in the laboratory. All five contain SPs and were observed to interact with RBC membranes [21]. The motivation for our study was the expectation that this list may be incomplete. We propose four possible reasons. First, the Gohil study uses only a signal-based approach and discards proteins without SPs. Second, only one SP prediction program is used, SignalP, and whether to discard or keep a protein is determined by comparing an output score to a user defined threshold (i.e., a rule-based decision). Similarly, only one TM and one GPI program are used with applied thresholds. Third, an unknown percentage of the SP, TM, and GPI predictions will be incorrect due to the inherent imprecise nature of all prediction programs; and fourth, no other study has verified the reported exportome. We applied the rule-based selection criteria to the training file using the same Gohil prediction programs but with a newer version of SignalP (PredGPI was also used instead of DGPI); and 4.8% positives and 14.9% negatives were classified differently to that expected. The expectation is that there should be no false predictions given that the training file is equivalent to the exportome predicted in the Gohil study. The current study builds upon the work by Gohil and colleagues and presents three ML methods to predict exportome membership.

There is currently no laboratory verified list of exportome proteins for *B. bovis* or even for closely related species *B. bigemina* and *B. canis*. This presented an unavoidable challenge to the study in that there are no verified data for ML training or validating the predictions. The reported Gohil exportome proteins therefore served as example targets (i.e., positives) for our study due to the absence of an alternative examples source. However, all the examples contain SPs, which presented an inescapable bias to our study. For example, not all secreted proteins have SPs (i.e., non-classical pathway proteins) [43,50], although non-classical pathway *Babesia* proteins associated with RBC membranes are yet to be reported. Moreover, SP-containing proteins are known to be involved in both RBC invasion and modification.

Sequences of non-secretory proteins were considered of equal informative importance to target sequences. That is, sequence differences provide the important information for ML classification. For example, frequency counts of single amino acids, dipeptides, tripeptides and quad-peptides were compared between target (positive) and non-secretory (negative) protein sequences within the first 25, end 25, and the remaining centre amino acids. The comparisons clearly showed observable differences between positives and negatives at the start and end. Nonetheless, there was no clear consensus to apply a rule-based approach to accurately distinguish between positives and negatives. For example, VA is the most commonly occurring dipeptide in the first 25 amino acids of positives (68% VA presence as opposed to 8% in negatives). Classifying proteins based on a dipeptide presence or absence rule would therefore be inaccurate, e.g., not all positives contain a VA. Conversely, the ML applied here had the capacity to detect distinguishing sequence patterns between the positives and negatives as demonstrated with Method #1 using dipeptides (90.89% accuracy). No simple sequence consensus exists and this is assumed to be due to sequence patterns evolving over time.

The premise for Method #2 is that although sequence patterns evolve, exportome proteins must still retain the same functionality. PSSM attempts to capture the inherent variability of sequence patterns by aligning a set of homologous sequences (conserved positions have higher scores than variable positions). We assumed here that the positive, as opposed to negative, homologous sequences had a functional relationship. The crucial part to this study was how to create training data for ML from the PSSM. The Verma Study [24] generated a 400-dimensional input vector by summing up all rows in the PSSM corresponding to the same amino acid in the primary sequence. Then, every element in this input vector was divided by the length of the sequence and scaled to the range of 0–1 by using a standard sigmoid function. For example, 400 features labelled AA, AC, AD, AE, etc., where ‘AA’ contained the sum of the ‘A’ column for all rows with primary sequence letter ‘A’; and likewise, AC contained the sum of the ‘C’ column for all rows with primary sequence letter ‘A’, etc. We followed this Verma methodology but trained the models with all six ML algorithms. SVM had the best accuracy with 83.41% from 10-fold cross validation (see Supplementary Table S4—Method #1). Interestingly, the Verma study only used SVM. The Verma methodology does not take into account amino acid position in the protein sequence. As was shown in Supplementary Table S2, the occurrence of particular amino acids at set positions within the first 25 amino acids of the positive sequences is not random. Our methodology for creating training data from the PSSM takes into account amino acid positions. The accuracy achieved as per Method #2 was 89.95%, hence we propose that our methodology is a slight improvement to the one described by Verma.

Six prediction programs were used in Method #3 to predict protein characteristics associated with protein localization. All programs were amino acid sequence-based, focusing on delivery signals and/or amino acid composition using rules-based and/or ML: SignalP (SPs via ML); WoLF PSORT (delivery signals and amino acid composition via both rules-based and ML), TargetP (SPs via ML); TMHMM (TMs via ML); Phobius (SPs and TMs via ML); and DeepLoc 1.0 (delivery signals and amino acid composition via ML). A point previously raised was that all prediction programs can be expected to have an unknown percentage of inaccurate predictions. This point was shown to be factual. For example, the Gohil study used SignalP version 3.0, which has subsequently been updated to 5.0. In our study, 35 out of the 214 positive proteins had a score less than 0.5, which indicates no SP. The aim of Method #3 was to determine if the collection of predicted protein characteristics contained a pattern that could be exploited by ML for classification, irrespective of inherent inaccuracies in source predictions. For example, if classification was based on the SignalP score alone, 35 out of 214 positive proteins would be misclassified given a 0.5 threshold. Similarly, 8, 23, 37, 120 would be misclassified using scores from Phobius, TargetP, WoLF PSORT, and DeepLoc, respectively. However, only four proteins were misclassified when using ML with the protein characteristics collection as per Method #3.

Ten-fold cross validation was performed on reduced numbers of features (100, 200, 300, and 800 or 1000) to assess the possibility the large numbers of features in Methods #1 and #2 compared to the small number of examples may lead to an unwanted overfit solution. We conclude that using an ensemble of classifiers is a reasonable solution to accommodate the presented ratio of examples and features given the observed marginal difference in accuracy when using a small or large number of features. Moreover, 10-fold cross validation and train–validation–test results from all three described methods clearly demonstrated that ML could accurately perform classification on the positive and negative datasets. The challenge is that the definite pattern(s) determining the classifications remains an unknown entity, which is a common occurrence in ML projects due to their mostly ‘black-box’ nature. For example, we do not know if the ML algorithms were finding patterns specific to known characteristics of the training sequences (e.g., presence of SPs) or novel patterns associated with actual exportome proteins or a combination of both. To help resolve this latter conundrum, we applied all three methods to 25 PfEMP1 proteins, which lack a SP and contain TM domains. Not one PfEMP1 protein was predicted by the three methods to be an exportome member. Although based on only one type of non-classical protein, this result suggests that the ML algorithms are only finding patterns specific to proteins respecting the conventional secretory pathway. This was not completely unexpected (especially for Method #3) because the algorithms were of course trained on these types of proteins. Conversely, it was still speculated at the onset of this study that there may be additional exclusive sequence signals and/or amino acid compositions common to only exportome proteins, which Methods #1 and #2 might detect. This speculation developed because intuitively there must be a particular determinant why only *some* secretory proteins interact with RBC membranes. The premise here was that the determinant might be encoded in the primary protein sequence, but it is equally probable there are no exclusive signals to find. For example, the determinant may only be revealed by studying secondary and/or tertiary structures, or even a yet to be discovered protein localization mechanism.

Unified SecretomeP and OutCyte scores were used to capture non-classical exportome proteins missed by the three methods. However, OutCyte and SecretomeP have many contradictory predictions, which remain a concern.

Many exported *Plasmodium* proteins have a PEXEL motif. Results from the current study showed that a PEXEL plays an inconsequential part, if any, in the export of *Babesia* proteins. However, a question that arises is whether there are one, two or more contributing signals encoded in protein sequences that guide a newly synthesised protein from within the parasite to the RBC membrane. For example, a *Plasmodium* export protein encounters three physical barriers: a parasite membrane (PM), a parasitophorous vacuole membrane (PVM), and the RBC membrane (either to transport through or on to the surface). With respect to the classical secretory pathway, the literature suggests there are at least two signals directing a *Plasmodium* protein through the barriers: an SP for the PM, and a PEXEL motif downstream of the SP for the PVM. It is unclear whether the remaining mature sequence after the SP and PEXEL cleavage sites also encodes a signal for further direction, i.e., what determines why some proteins remain in the RBC cytosol whilst others interact with the RBC membrane. Note that some known *P. falciparum* exported proteins such as KAHRP, GBP130 protein, and RESA have no SP predicted by SignalP 5.0. This suggests that there are other signals, possibly recessed SPs, targeting these export proteins through the PM.

We propose that it is likely that *Babesia* has more than one signal directing an exported protein (although PVM is not a barrier as it disappears within minutes of invading the RBC [69]). Methods #1 and #2 use 25 amino acids (AAs) at the start of a sequence, a region SPs are typically located. It is possible these two methods are exploiting patterns exemplifying a SP as a discriminator between positives and negatives rather than a pattern that is unique to an exportome protein. This would mean the methods only predict (albeit with reasonable accuracy) proteins escaping the PM. However, 73 (22%) of the 337 predicted *Babesia* exportome proteins do not have a predicted SP, and 13 SP-ontaining proteins were not predicted as exportome members. During the initial testing of the methods, 30 and 40 AAs at the start and end of a sequence were also evaluated but had worse results than 25 AAs in 10-fold cross validation. This suggests that an additional export signal, if one exists, would be more than 40 AAs from the N-terminal.

A proposal for future research when many more verified exportome proteins are known is to use an equal proportion of SP-containing proteins in both positives and negatives. For example, positives would consist of known exportome proteins, where a proportion is expected to have SPs; and negatives would mainly be those proteins with known SPs that invade RBCs. Given ML training data with a proportional number of SPs, a specific exportome signal should be unambiguously detectable, if one existed.

A further challenge to the study was the uncertainty in the annotation quality of protein names. UniProtKB [70] provides a heuristic measure of the annotation, although the curators claim they cannot define the ‘correct annotation’ for any given protein (https://www.uniprot.org/help/annotation_score [Date last accessed: 27 May 2021]). UniProtKB have assigned an annotation score from one to five to every protein, where five is considered the best-annotated entry (annotations with experimental evidence score higher than equivalent predicted/inferred annotations). With an understanding UniProtKB annotation scores are only a guideline of annotation quality, we checked scores for all *B. bovis* proteins: 89.1% scored 1, 10% scored 2, 0.87% scored 3, and 0.03% scored 4. The poor annotation had two implications. First, protein names appeared to contradict expectations following the selection of the training data based on the rule-based approach. For example, VESA named proteins were in both negative and positive datasets. Second, appraising the prediction methods based on protein names is potentially inaccurate given the poor annotation. Consequently, protein sequences took precedence over names in this study, despite sequences having their own levels of inaccuracies. The high-scoring exportome proteins presented in the results must therefore come with a caveat that their names may be misleading with regard to their sequence signals encoded and true function.

Comparison between exportome predictions of the three *Babesia* species clearly showed differences between them at the sequence level, i.e., some exportome proteins have sequences in all three species with >75% similarity (common proteins), whilst others have <25% sequence similarity (unique proteins). The only named high-scoring proteins that were common to all three species were heat shock proteins (HSP70 and HSP90). There is no evidence that *Babesia* heat shock proteins have any association with RBC modifications or even an association with DnaJ proteins in the RBC cytoplasm. However, there are publications debating the role of *P. falciparum* HSP70. A 2002 study [71] reports that the *P. falciparum* HSP70 is restricted to the parasite cytoplasm, suggesting that exported DnaJ molecules interact with the RBC HSP70; whereas a 2012 study [72] provide evidence for the trafficking of a parasite-encoded HSP70 into the RBC. We propose that common proteins are likely to have a role in altering the RBC and are worthwhile targets. Furthermore, we speculate that the unique *B. bovis* exportome proteins contribute to the ridge-like structures and are therefore influencing factors towards the increased virulence over *B. bigemina*.

## 4. Materials and Methods

Each method (dipeptide amino acid composition, position-specific scoring matrix, and subcellular location) is implemented by three independent configurable pipelines of linked bioinformatics programs, Python and Perl scripts, R functions and Linux shell scripts. The pipelines are designed to facilitate an automated, high-throughput computational approach to predict exportome proteins. The underlying premise of each method is that a difference exists between the sequences or characteristics defining exportome (positives) to those of non-secretory (negatives) proteins. A rule-based approach was not feasible because the differences were not consistently observed to accurately classify proteins. Conversely, ML has the capacity to detect obscure differences. The pipelines therefore included an ensemble of ML algorithms trained on known T2Bo positives and negatives to classify proteins as exportome or non-exportome based on a classification probability. A ReadMe file outlining the steps to run each pipeline is included with the source code (available at https://github.com/goodswen/exportome [Date last accessed: 27 May 2021]). The pipelines were designed for a Linux operating system and have only been tested on Red Hat Enterprise Linux 7.7, but are expected to work on most Linux distributions.

### 4.1. Data Source

All 3706 currently available protein sequences for *B. bovis* T2Bo were downloaded in a FASTA format from PiroPlasmaDB (release 47), which is a database member of Eukaryotic Pathogen Databases (EuPathDB) [73]. Similarly, all 5077 protein sequences for *B. bigemina* BOND were downloaded from PiroPlasmaDB. Protein sequences for 3467 *B. canis* BcH-CHIPZ were extracted from a Supplementary Excel spreadsheet created from the *B. canis* genome sequencing study [2]. Sequences from all three species were used in a FASTA format as primary input for each of the three methods.

Sequences for all 5460 *P. falciparum* (strain 3D7) and 8322 *T. gondii* (strain ME49) proteins were downloaded in a FASTA format from PlasmoDB (release 47) and ToxoDB (release 47), respectively, which are database members of EuPathDB. The 196 *P. falciparum* proteins listed in PlasmodDB as exported proteins were predicted by a program called ExportPred [25]. This program uses a generalised hidden Markov model (GHMM) to model simultaneously the SP sequence and PEXEL motif features.

### 4.2. Training Input Sequences for Machine Learning

Sequences of 214 proteins reported in the Gohil study [21] as belonging to the *B. bovis* T2Bo exportome were extracted from the 3706 total to form a ‘positives’ dataset. That is, these sequences possess a SP but no TM domains and GPI motifs. The Gohil study also classified 3286 *B. bovis* proteins as ‘non-secretory’ (classified mainly by selecting proteins with no predicted SP but with TMs and/or GPI anchors)—214 sequences were randomly selected from the 3286 non-secretory proteins using the Python random module to form a ‘negatives’ dataset. The ML input training file for each method therefore consisted of 428 sequences representing the positives and negatives datasets. Supplementary Table S7, sheet ‘Training_proteins’ lists the proteins that were used for training.

The program CD-HIT (cluster database at high identity with tolerance) [74] was used to determine whether any of the training sequences had 100% similarity (i.e., a check for redundant sequences). Two identical clusters were detected for positives (BBOV_IV009870, BBOV_IV009860 and BBOV_III006480, BBOV_III006480). A further seven clusters of positive proteins had similarities >90% (see Data S1). These proteins were assumed to be isoforms rather than the same proteins incorrectly assigned with unique IDs. No identical sequences were detected for negatives. The training data provided in the publicly available pipelines do not include four negatives that were consistently misclassified during the 10-fold cross validation, and two identical positives detected by CD-HIT.

### 4.3. Machine Learning Algorithms

Six supervised machine learning (ML) algorithms were used via R functions to build predictive models: (1) adaptive boosting (AdaBoost) [75] via the *ada* R function [76]; (2) random forest (RF) [77] via the *randomForest* R function; (3) *k*-nearest neighbour classifier (*k*NN) [78] via a *knn R* function contained in the *Class* package; (4) naive Bayes classifier (NB) [79] via a *naiveBayes R* function contained in the *e1071* package; (5) neural network (ANN) [80] via the *nnet* R function contained in the *nnet* package; and (6) support vector machines (SVM) [81] via the *ksvm* R function, which is contained in the *kernlab* package. All ML R functions used at least two arguments: a data frame of numeric variables (i.e., a training dataset) and a numerical class vector, i.e., a vector representing the target label, which had two classes: 1 (positive) and 0 (negative).

Each algorithm generates a probability that a classification is correct. All three methods compared these classification probabilities to a threshold value (0.5) to determine the relevant class, positive (exportome) or negative (non-exportome). Furthermore, the algorithms were used in different combinations as an ensemble of classifiers, i.e., classification probabilities from each algorithm in the ensemble were averaged to determine the final classification probability.

### 4.4. Method #1—Dipeptide Amino Acid Composition

There are 400 possible dipeptide combinations (i.e., 20 possible amino acids raised to the power of two consecutive amino acids, e.g., combinations of AA, AR, AN … RA, NA (see Figure 2)). The training data consisted of 800 columns (features/predictors) representing dipeptide amino acid counts of 400 dipeptide combinations at the start (N-terminus) and 400 at the end (C-terminus) of the training input sequences. No feature scaling (i.e., normalisation, standardisation) was applied because all features had the same source and therefore had the same degrees of magnitude, range, and units.

### 4.5. Method #2—Position-Specific Scoring Matrix (PSSM)

The position-specific iterated BLAST (PSI-BLAST) program [82] was used to generate a set of aligned sequences and create a single PSSM for each training file protein. PSI-BLAST search was performed against the ‘nr’ database using 3 iterations with a 0.001 e-value. Figure 3 shows an extract of a PSSM for one protein. There are 20 columns for each possible amino acid and one row for each amino acid in the primary protein sequence, e.g., the protein in Figure 3 starts with the letters ‘MKK’. Values in the matrix are a normalised (log likelihood) frequency count of each amino acid (20 in total) at the same position in the PSI-BLAST aligned sequences, e.g., the scores are A = −1, R = −1, etc., for position #1 containing the primary sequence M.

The first and last 25 amino acids of the primary protein sequence were used from the PSSM to create the training file. There were 1000 features labelled P1A, P1R, P1N, P1D, etc., representing PSSM scores for the 20 amino acids for position #1 (‘P’ is the position prefix) in the primary sequence, and then P2A, P2R, P2N, P2D, etc., for position #2, etc. In the Figure 3 example, P1A = −1 and P1R = −1. Similarly to Method #1, no feature scaling was applied.

### 4.6. Method #3—Subcellular Location

Six programs were used to predict various protein characteristics encoded within the training protein sequences: SignalP 5.0 [56] (predicts presence and location of signal peptide cleavage sites using deep neural networks); WoLF PSORT 0.2 and TargetP 1.1 [43] (predict subcellular localization); TMHMM 2.0 [53] (predicts transmembrane domains in proteins); Phobius 1.0 [57] (predicts transmembrane topology and signal peptides); and DeepLoc 1.0 [58] (predicts eukaryotic protein subcellular localization using deep learning). The prediction methods can be broadly grouped into two classification types: rules-based and ML. The rules-based method exploits static knowledge of what determines subcellular location, whereas ML predicts subcellular locations by focusing on differences between proteins from different known locations [43].

There were 11 features extracted from the six programs, which represented a mixture of data types corresponding to an accuracy measure, a perceived reliability, or a type of score for the protein characteristic being predicted (see ReadMe in Supplementary Table S5). In summary, the training data consisted of 428 rows by 11 columns representing predicted protein characteristics for the positive and negative datasets. Normalisation or standardisation was applied to the six features (phobius_TM, tmhmm_ExpAA, tmhmm_First60, tmhmm_PredHel, wolf_score, wolf_annotation) that do not have a 0 to 1 range, and the impact to the ML performances compared. The formulae used were normalised *x* = (*x* − minimum *x*)/(maximum *x* − minimum *x*) and standardised *x* = (*x* − μ)/σ, where ‘*x*’ is the feature value, and minimum and maximum relates to the minimum or maximum feature value when considering the entire dataset and ‘μ’ is the sample mean and ‘σ’ is the standard deviation.

### 4.7. Validation of Training Data

Performances of the ML models were evaluated using 10-fold cross validation and 100 times train (70%)–validate (15%)–test (15%) splits on the training data. The performance measures used were accuracy, error rate, sensitivity, false-positive rate, specificity, positive and negative predictive values (see ReadMe in Supplementary Table S3 for formulae).

### 4.8. Predicting Non-Classical Exported Proteins

SecretomeP [50] and OutCyte [51] were used to predict non-classical secreted proteins given 3706 *B. bovis* T2Bo, 5077 *B. bigemina* BOND and 3467 *B. canis* BcH-CHIPZ proteins. A protein was considered non-classical if the OutCyte class was ‘unconventional protein secretion’ (UPS) and the SecretomeP score was greater or equal to 0.6 (a threshold recommended by the SecretomeP creators) and SignalP 5.0 [56] score was less than or equal to 0.5 (i.e., no signal peptide).

### 4.9. Plasmodium PEXEL Motifs

The *Plasmodium* PEXEL motif consists of R.L.[EQD], where [EQD] means E, Q or D can occur and ‘.’ means any amino acid for the respective position [67]. Both soluble and membrane-embedded proteins can contain a PEXEL [61]. One review [61] states the motif is typically located at approximately 35 amino acids downstream of a signal sequence whilst another review [83] suggests 20–35. An in-house Python script determined the location of the first PEXEL motif when searching from N to C terminals on all *P. falciparum* 3D7, *T. gondii* ME49 and *B. bovis* T2Bo protein sequences.

### 4.10. Method Implementation

The presented three methods have been implemented in three Linux pipelines. These pipelines consist of linked Python and Linux shell scripts, and R functions. The pipelines are designed to facilitate an automated, high-throughput computational approach to predict exportome membership probabilities. All three pipelines were designed for a Linux operating system and have only been tested on Red Hat Enterprise Linux 7.7, but are expected to work on most Linux distributions. They are freely available at: https://github.com/goodswen/exportome [Date last accessed: 27 May 2021] (see ReadMe.txt for pipeline instructions).

## 5. Conclusions

In the current study, the three described methods assigned a probability score expressing exportome membership to all known *B. bovis* T2Bo (minus those used for ML training), *B. bigemina* BOND, and *B. canis* BcH-CHIPZ proteins. Based on 10-fold cross validation and multiple train–validation–test splits of training data, we expect that over 90% of these scores accurately provide a secretory (via a conventional pathway) or non-secretory indicator, i.e., an indicator that the protein escapes the parasite membrane into the RBC cytosol. However, only laboratory testing can verify that the highest scoring are indeed exported proteins. Furthermore, until there are more experimentally proved exportome proteins, we cannot report with any certainty whether primary protein sequences encode information that distinguishes those proteins destined for the host RBC from other secretory proteins. Our expectation is that ML will have the capacity to clear this uncertainty given training data with many more proved examples. The presented methods, as is the initial case with all ML projects, require iterative cycles of ML predictions, laboratory feedback and training data adjustment. This is especially true in the case for *B. bovis*, with limited experimental knowledge of what truly constitutes effective therapeutic candidates against babesiosis. The current three methods at least provide those proteins most worthy of laboratory validation to initiate this required cyclic approach. We make available to the public these methods implemented in Linux pipelines. The pipelines are designed to facilitate an automated, high-throughput computational approach and can be used or adapted by other researchers to predict an exportome for their intracellular parasite of interest.

## Figures and Tables

**Table 1:** Performance measures for method #1—dipeptide amino acid composition.

Algorithm	Accuracy (%)	Error Rate (%)	Sensitivity (%)	False Positive Rate (%)	Specificity (%)	Positive Predictive Value (%)	Negative Predictive Value (%)
Ensemble	90.89	9.11	91.12	9.35	90.65	90.7	91.08
SVM	90.19	9.81	90.65	10.28	89.72	89.81	90.57
adaBoost	89.49	10.51	88.79	9.81	90.19	90.05	88.94
RF	89.25	10.75	91.59	13.08	86.92	87.5	91.18
ANN	87.15	12.85	87.38	13.08	86.92	86.98	87.32

Note: ANN = artificial neural network; RF = random forest; SVM = support vector machine; Ensemble = SVM, adBoost, RF, and ANN.

**Table 2:** Performance measures for Method #2—position-specific scoring matrix (PSSM).

Algorithm	Accuracy (%)	Error Rate (%)	Sensitivity (%)	False Positive Rate (%)	Specificity (%)	Positive Predictive Value (%)	Negative Predictive Value (%)
RF	89.95	10.05	88.79	8.88	91.12	90.91	89.04
adaBoost	89.25	10.75	88.79	10.28	89.72	89.62	88.89
Ensemble	88.32	11.68	87.85	11.21	88.79	88.68	87.96
SVM	88.08	11.92	87.85	11.68	88.32	88.26	87.91
ANN	84.58	15.42	85.05	15.89	84.11	84.26	84.91

Note: ANN = artificial neural network; RF = random forest; SVM = support vector machine; Ensemble = SVM, adBoost, RF, and ANN.

**Table 3:** Performance measures for Method #3—subcellular prediction.

Algorithm	Accuracy (%)	Error Rate (%)	Sensitivity (%)	False Positive Rate (%)	Specificity (%)	Positive Predictive Value (%)	Negative Predictive Value (%)
Ensemble	96.26	3.74	98.13	5.61	94.39	94.59	98.06
RF	95.56	4.44	97.66	6.54	93.46	93.72	97.56
SVM	95.09	4.91	96.73	6.54	93.46	93.67	96.62
adaBoost	94.86	5.14	96.26	6.54	93.46	93.64	96.15
NB	93.46	6.54	96.26	9.35	90.65	91.15	96.04
ANN	93.22	6.78	92.99	6.54	93.46	93.43	93.02
kNN	89.72	10.28	90.19	10.75	89.25	89.35	90.09

Note: ANN = artificial neural network; kNN = *k*- nearest neighbour classifier; NB = naive Bayes classifier; RF = random forest; SVM = support vector machine; Ensemble = adBoost, ANN, kNN, NB; RF, and SVM.

## Data Availability

Source code provided at: https://github.com/goodswen/exportome (Date last accessed: 27 May 2021).

## Supplementary Materials

The following are available online at https://www.mdpi.com/article/10.3390/pathogens10060660/s1, Table S1: Results from rule-based approach for predicting an exportome and results from amino acid frequency analysis; Table S2: Results from amino acid frequency analysis at specific locations at start and end of proteins; Table S3: Results from Method #1—dipeptide amino acid composition; Table S4: Results from Method #2—position-specific scoring matrix (PSSM); Table S5: Results from Method #3—subcellular location; Table S6: Comparison of classification outcomes from the three exportome prediction methods; Table S7: Full *Babesia bovis* T2Bo exportome prediction; Table S8: Full *Babesia bigemina* BOND exportome prediction; Table S9: Full *Babesia canis* BcH-CHIPZ exportome prediction; Table S10: Predicted non-classical exported proteins; Table S11: Results from benchmarking the three exportome prediction methods against *Plasmodium falciparum* and *Toxoplasma gondii*.
