# Supplementary material for: Applying Machine Learning to Predict the Exportome of Bovine and Canine Babesia Species That Cause Babesiosis
Source: Pathogens. 2021 May 27;10(6):660. doi: 10.3390/pathogens10060660 (PMC8226867; doi:10.3390/pathogens10060660)
Supplement: Supplementary file 1 [file pathogens-10-00660-s001.zip › Supplementatry_files/Data_S1.pdf]

Applying Machine Learning to Predict the Exportome of Bovine and Canine *Babesia* Species that Cause Babesiosis (Stephen J. Goodswen, Paul J. Kennedy and John T. Ellis)

Correspondence: John.Ellis@uts.edu.au

CD-HIT results

### Positive (exportome) proteins

Program: CD-HIT, V4.8.1 (+OpenMP)

Command: ./cd-hit -i positives_214.fa -o cdhit_positives_214

Started: 17:21:10 2021

==

Output

total seq: 214

longest and shortest : 1089 and 59

Total letters: 63660

Sequences have been sorted

Approximated minimal memory consumption:

Sequence : 0M

Buffer : 1 X 10M = 10M

Table : 1 X 65M = 65M

Miscellaneous : 0M

Total : 76M

Table limit with the given memory limit:

Max number of representatives: 1644743

Max number of word counting entries: 90479180

comparing sequences from 0 to 214

214 finished   204 clusters

Approximated maximum memory consumption: 76M

writing new database

writing clustering information

program completed !

Total CPU time 0.07

==

>Cluster 0

0 1089aa, >BBOV_I004210... *

>Cluster 1

0 865aa, >BBOV_III003260... *

>Cluster 2

0 814aa, >BBOV_III004200... *

>Cluster 3

0 795aa, >BBOV_III007380... *

>Cluster 4

0 784aa, >BBOV_IV002270... *

>Cluster 5

0 781aa, >BBOV_IV007620... *

>Cluster 6

0 778aa, >BBOV_IV009240... *

>Cluster 7

0 665aa, >BBOV_III005960... *

>Cluster 8

0 652aa, >BBOV_III007800... *

>Cluster 9

0 651aa, >BBOV_IV007010... *

>Cluster 10

0 627aa, >BBOV_III001600... *

>Cluster 11

0 622aa, >BBOV_IV004810... *

>Cluster 12

0 613aa, >BBOV_II004280... *

>Cluster 13

0 613aa, >BBOV_III004080... *

>Cluster 14

0 593aa, >BBOV_II002190... *

>Cluster 15

0 574aa, >BBOV_II002880... *

>Cluster 16

0 565aa, >BBOV_IV009870... *

1 565aa, >BBOV_IV009860... at 100.00%

>Cluster 17

0 559aa, >BBOV_II001970... *

>Cluster 18

0 548aa, >BBOV_III005180... *

>Cluster 19

0 531aa, >BBOV_IV001070... *

>Cluster 20

0 517aa, >BBOV_IV007750... *

>Cluster 21

0 510aa, >BBOV_II004730... *

>Cluster 22

0 509aa, >BBOV_III009440... *

>Cluster 23

0 501aa, >BBOV_I000160... *

>Cluster 24

0 478aa, >BBOV_II007340... *

>Cluster 25

0 473aa, >BBOV_III001500... *

>Cluster 26

0 463aa, >BBOV_IV002330... *

>Cluster 27

0 459aa, >BBOV_IV006390... *

>Cluster 28

0 438aa, >BBOV_IV003980... *

>Cluster 29

0 437aa, >BBOV_III006470... *

1 437aa, >BBOV_III006510... at 98.86%

2 437aa, >BBOV_III006490... at 99.77%

>Cluster 30

0 437aa, >BBOV_III005800... *

1 437aa, >BBOV_III005620... at 94.05%

>Cluster 31

0 433aa, >BBOV_III008960... *

>Cluster 32

0 432aa, >BBOV_III011590... *

>Cluster 33

0 424aa, >BBOV_III011350... *

>Cluster 34

0 423aa, >BBOV_III007790... *

>Cluster 35

0 423aa, >BBOV_IV003350... *

>Cluster 36

0 419aa, >BBOV_III001660... *

>Cluster 37

0 418aa, >BBOV_II000720... *

>Cluster 38

0	416aa, >BBOV_IV003320... *

>Cluster 39

0	414aa, >BBOV_III006100... *

>Cluster 40

0	411aa, >BBOV_III011330... *

>Cluster 41

0	404aa, >BBOV_I000820... *

>Cluster 42

0	395aa, >BBOV_IV009100... *

>Cluster 43

0	394aa, >BBOV_II003360... *

>Cluster 44

0	392aa, >BBOV_IV006420... *

>Cluster 45

0	373aa, >BBOV_IV001090... *

>Cluster 46

0	372aa, >BBOV_IV005390... *

>Cluster 47

0	371aa, >BBOV_I000970... *

>Cluster 48

0	361aa, >BBOV_I004780... *

>Cluster 49

0	358aa, >BBOV_II002280... *

>Cluster 50

0	354aa, >BBOV_I001420... *

>Cluster 51

0	354aa, >BBOV_IV004890... *

>Cluster 52

0	352aa, >BBOV_IV005440... *

>Cluster 53

0 351aa, >BBOV_IV004360... *

>Cluster 54

0 351aa, >BBOV_I002900... *

>Cluster 55

0 349aa, >BBOV_IV010280... *

>Cluster 56

0 346aa, >BBOV_IV008150... *

>Cluster 57

0 342aa, >BBOV_II002660... *

>Cluster 58

0 341aa, >BBOV_III003500... *

>Cluster 59

0 339aa, >BBOV_IV008060... *

>Cluster 60

0 339aa, >BBOV_II001640... *

>Cluster 61

0 323aa, >BBOV_III010990... *

>Cluster 62

0 323aa, >BBOV_III007330... *

>Cluster 63

0 312aa, >BBOV_IV005750... *

>Cluster 64

0 308aa, >BBOV_IV010790... *

>Cluster 65

0 306aa, >BBOV_II000710... *

>Cluster 66

0 300aa, >BBOV_IV003220... *

>Cluster 67

0 299aa, >BBOV_III007430... *

>Cluster 68

0 298aa, >BBOV_II002830... *

>Cluster 69

0 298aa, >BBOV_IV000670... *

>Cluster 70

0 295aa, >BBOV_III011930... *

>Cluster 71

0 294aa, >BBOV_III006630... *

>Cluster 72

0 293aa, >BBOV_III007520... *

>Cluster 73

0 292aa, >BBOV_I001180... *

>Cluster 74

0 292aa, >BBOV_I003890... *

>Cluster 75

0 291aa, >BBOV_IV008620... *

>Cluster 76

0 285aa, >BBOV_III006480... at 100.00%

1 291aa, >BBOV_III006500... *

>Cluster 77

0 290aa, >BBOV_I001090... *

>Cluster 78

0 290aa, >BBOV_III006460... *

>Cluster 79

0 286aa, >BBOV_III005630... *

>Cluster 80

0 285aa, >BBOV_III003290... *

>Cluster 81

0 283aa, >BBOV_III005600... *

>Cluster 82

0 283aa, >BBOV_IV007970... *

>Cluster 83

0 282aa, >BBOV_III005720... *

>Cluster 84

0 282aa, >BBOV_III002860... *

>Cluster 85

0 281aa, >BBOV_I004040... *

>Cluster 86

0 280aa, >BBOV_III009100... *

>Cluster 87

0 277aa, >BBOV_III000800... *

>Cluster 88

0 276aa, >BBOV_IV001030... *

>Cluster 89

0 274aa, >BBOV_IV006500... *

>Cluster 90

0 274aa, >BBOV_IV006480... *

>Cluster 91

0 274aa, >BBOV_II002820... *

>Cluster 92

0 273aa, >BBOV_IV002450... *

>Cluster 93

0 272aa, >BBOV_I001050... *

>Cluster 94

0 272aa, >BBOV_I001120... *

>Cluster 95

0 271aa, >BBOV_IV005650... *

>Cluster 96

0 271aa, >BBOV_III006540... *

>Cluster 97

0 269aa, >BBOV_IV000860... *

>Cluster 98

0 268aa, >BBOV_III011700... *

>Cluster 99

0 267aa, >BBOV_I003500... *

>Cluster 100

0 265aa, >BBOV_I001680... *

>Cluster 101

0 265aa, >BBOV_I001670... *

>Cluster 102

0 264aa, >BBOV_II004150... *

>Cluster 103

0 262aa, >BBOV_II004430... *

>Cluster 104

0 259aa, >BBOV_IV002930... *

>Cluster 105

0 257aa, >BBOV_II001380... *

>Cluster 106

0 256aa, >BBOV_II007300... *

>Cluster 107

0 256aa, >BBOV_III002340... *

>Cluster 108

0 255aa, >BBOV_IV004340... *

>Cluster 109

0 253aa, >BBOV_II005290... *

>Cluster 110

0 253aa, >BBOV_III000390... *

>Cluster 111

0 252aa, >BBOV_II002580... *

>Cluster 112

0 250aa, >BBOV_IV000770... *

>Cluster 113

0 249aa, >BBOV_IV010000... *

>Cluster 114

0 244aa, >BBOV_II002570... *

>Cluster 115

0 241aa, >BBOV_IV008100... *

>Cluster 116

0 241aa, >BBOV_II006810... *

>Cluster 117

0 241aa, >BBOV_IV007930... *

>Cluster 118

0 236aa, >BBOV_III006520... *

>Cluster 119

0 236aa, >BBOV_IV009170... *

>Cluster 120

0 235aa, >BBOV_II000400... *

>Cluster 121

0 235aa, >BBOV_II003960... *

>Cluster 122

0 234aa, >BBOV_IV011310... *

>Cluster 123

0 233aa, >BBOV_IV003120... *

>Cluster 124

0 233aa, >BBOV_II002590... *

>Cluster 125

0 233aa, >BBOV_II000060... *

1 233aa, >BBOV_III000050... at 92.27%

>Cluster 126

0 231aa, >BBOV_IV000780... *

>Cluster 127

0 228aa, >BBOV_IV008970... *

>Cluster 128

0 226aa, >BBOV_IV000420... *

>Cluster 129

0 220aa, >BBOV_III009330... *

>Cluster 130

0 217aa, >BBOV_IV005000... *

>Cluster 131

0 217aa, >BBOV_I004270... *

>Cluster 132

0 217aa, >BBOV_IV004250... *

>Cluster 133

0 211aa, >BBOV_II004220... *

>Cluster 134

0 211aa, >BBOV_I002480... *

>Cluster 135

0 211aa, >BBOV_I001020... *

>Cluster 136

0 208aa, >BBOV_III005830... *

>Cluster 137

0 208aa, >BBOV_IV011670... *

>Cluster 138

0 204aa, >BBOV_IV008610... *

>Cluster 139

0 203aa, >BBOV_II001760... *

>Cluster 140

0 202aa, >BBOV_IV005180... *

>Cluster 141

0 197aa, >BBOV_I003150... *

>Cluster 142

0 196aa, >BBOV_IV012140... *

1 196aa, >BBOV_IV000040... at 91.84%

>Cluster 143

0 195aa, >BBOV_III010940... *

>Cluster 144

0 192aa, >BBOV_IV007960... *

>Cluster 145

0 192aa, >BBOV_II000990... *

>Cluster 146

0 191aa, >BBOV_IV000760... *

>Cluster 147

0 189aa, >BBOV_I001370... *

>Cluster 148

0 188aa, >BBOV_I004140... *

>Cluster 149

0 188aa, >BBOV_IV009060... *

>Cluster 150

0 188aa, >BBOV_II001390... *

>Cluster 151

0 188aa, >BBOV_II006740... *

>Cluster 152

0 187aa, >BBOV_III011960... *

1 187aa, >BBOV_I005150... at 94.65%

>Cluster 153

0 187aa, >BBOV_I001160... *

>Cluster 154

0 186aa, >BBOV_III007740... *

>Cluster 155

0 183aa, >BBOV_I001900... *

>Cluster 156

0	182aa, >BBOV_I001170... *

>Cluster 157

0	182aa, >BBOV_I000840... *

>Cluster 158

0	180aa, >BBOV_III011870... *

>Cluster 159

0	178aa, >BBOV_IV001040... *

>Cluster 160

0	178aa, >BBOV_IV005120... *

>Cluster 161

0	176aa, >BBOV_II000730... *

>Cluster 162

0	175aa, >BBOV_IV006290... *

>Cluster 163

0	173aa, >BBOV_I001070... *

>Cluster 164

0	172aa, >BBOV_IV011840... *

>Cluster 165

0	171aa, >BBOV_II005080... *

>Cluster 166

0	163aa, >BBOV_III010050... *

>Cluster 167

0	162aa, >BBOV_III005590... *

>Cluster 168

0	161aa, >BBOV_IV003250... *

>Cluster 169

0	159aa, >BBOV_III003930... *

>Cluster 170

0	159aa, >BBOV_I001130... *

>Cluster 171

0	156aa, >BBOV_IV000410... *

>Cluster 172

0	156aa, >BBOV_III000020... *

1	156aa, >BBOV_II007820... at 92.31%

>Cluster 173

0	152aa, >BBOV_III002240... *

>Cluster 174

0	150aa, >BBOV_I004860... *

>Cluster 175

0	143aa, >BBOV_I002420... *

>Cluster 176

0	142aa, >BBOV_III002350... *

>Cluster 177

0	142aa, >BBOV_III000690... *

>Cluster 178

0	142aa, >BBOV_II006800... *

>Cluster 179

0	140aa, >BBOV_IV005880... *

>Cluster 180

0	140aa, >BBOV_II007780... *

>Cluster 181

0	140aa, >BBOV_IV000090... *

>Cluster 182

0	139aa, >BBOV_IV001410... *

>Cluster 183

0	138aa, >BBOV_I003860... *

1	138aa, >BBOV_II004160... at 92.75%

>Cluster 184

0	135aa, >BBOV_III001320... *

>Cluster 185

0 133aa, >BBOV_III009090... *

>Cluster 186

0 132aa, >BBOV_IV007940... *

>Cluster 187

0 127aa, >BBOV_II002290... *

>Cluster 188

0 123aa, >BBOV_IV006430... *

>Cluster 189

0 117aa, >BBOV_IV003550... *

>Cluster 190

0 115aa, >BBOV_IV004940... *

>Cluster 191

0 115aa, >BBOV_IV004790... *

>Cluster 192

0 114aa, >BBOV_IV003470... *

>Cluster 193

0 109aa, >BBOV_II000510... *

>Cluster 194

0 108aa, >BBOV_III001690... *

>Cluster 195

0 108aa, >BBOV_IV008700... *

>Cluster 196

0 98aa, >BBOV_IV000070... *

>Cluster 197

0 91aa, >BBOV_II004780... *

>Cluster 198

0 87aa, >BBOV_IV009630... *

>Cluster 199

0 78aa, >BBOV_III002950... *

>Cluster 200

0	72aa, >BBOV_I001150... *

>Cluster 201

0	66aa, >BBOV_III000150... *

>Cluster 202

0	65aa, >BBOV_III001200... *

>Cluster 203

0	59aa, >BBOV_I005650... *

Negative (exportome) proteins

Program: CD-HIT, V4.8.1 (+OpenMP)

Command: ./cd-hit -i negatives_214.fa -o cdhit_negatives_214

Started: 17:19:13 2021

==

Output

----

total seq: 214

longest and shortest : 1821 and 51

Total letters: 96720

Sequences have been sorted

Approximated minimal memory consumption:

Sequence	: 0M

Buffer	: 1 X 10M = 10M

Table	: 1 X 65M = 65M

Miscellaneous	: 0M

Total	: 76M

Table limit with the given memory limit:

Max number of representatives: 1100062

Max number of word counting entries: 90455557

comparing sequences from   0 to   214

214 finished   214 clusters

Approximated maximum memory consumption: 77M

writing new database

writing clustering information

program completed !

Total CPU time 0.10

===

>Cluster 0

0	1821aa, >BBOV_IV009200... *

>Cluster 1

0	1651aa, >BBOV_IV011050... *

>Cluster 2

0	1603aa, >BBOV_II003570... *

>Cluster 3

0	1437aa, >BBOV_III004820... *

>Cluster 4

0	1385aa, >BBOV_III001280... *

>Cluster 5

0	1335aa, >BBOV_III002310... *

>Cluster 6

0	1323aa, >BBOV_III000650... *

>Cluster 7

0 1211aa, >BBOV_III007730... *

>Cluster 8

0 1151aa, >BBOV_I002970... *

>Cluster 9

0 1107aa, >BBOV_III000040... *

>Cluster 10

0 1102aa, >BBOV_IV007980... *

>Cluster 11

0 1099aa, >BBOV_I005780... *

>Cluster 12

0 1069aa, >BBOV_III009720... *

>Cluster 13

0 1040aa, >BBOV_III007250... *

>Cluster 14

0 1040aa, >BBOV_IV009720... *

>Cluster 15

0 1039aa, >BBOV_III003300... *

>Cluster 16

0 991aa, >BBOV_III005990... *

>Cluster 17

0 991aa, >BBOV_IV005090... *

>Cluster 18

0 991aa, >BBOV_I000250... *

>Cluster 19

0 988aa, >BBOV_II001010... *

>Cluster 20

0 959aa, >BBOV_III010710... *

>Cluster 21

0 957aa, >BBOV_IV011570... *

>Cluster 22

0 953aa, >BBOV_III008270... *

>Cluster 23

0 929aa, >BBOV_II004230... *

>Cluster 24

0 881aa, >BBOV_III009800... *

>Cluster 25

0 858aa, >BBOV_IV012020... *

>Cluster 26

0 846aa, >BBOV_IV005930... *

>Cluster 27

0 817aa, >BBOV_IV004710... *

>Cluster 28

0 815aa, >BBOV_II000760... *

>Cluster 29

0 810aa, >BBOV_IV004200... *

>Cluster 30

0 804aa, >BBOV_IV008360... *

>Cluster 31

0 790aa, >BBOV_I001080... *

>Cluster 32

0 781aa, >BBOV_II003580... *

>Cluster 33

0 750aa, >BBOV_III004910... *

>Cluster 34

0 731aa, >BBOV_IV001280... *

>Cluster 35

0 713aa, >BBOV_I004440... *

>Cluster 36

0 712aa, >BBOV_III004230... *

>Cluster 37

0 711aa, >BBOV_III003040... *

>Cluster 38

0 693aa, >BBOV_III003880... *

>Cluster 39

0 677aa, >BBOV_III009030... *

>Cluster 40

0 676aa, >BBOV_I001880... *

>Cluster 41

0 662aa, >BBOV_II007470... *

>Cluster 42

0 660aa, >BBOV_I004150... *

>Cluster 43

0 650aa, >BBOV_II006370... *

>Cluster 44

0 635aa, >BBOV_II002440... *

>Cluster 45

0 629aa, >BBOV_II004000... *

>Cluster 46

0 616aa, >BBOV_III009450... *

>Cluster 47

0 604aa, >BBOV_III007930... *

>Cluster 48

0 596aa, >BBOV_I002210... *

>Cluster 49

0 588aa, >BBOV_IV007780... *

>Cluster 50

0 571aa, >BBOV_III001400... *

>Cluster 51

0 561aa, >BBOV_II003730... *

>Cluster 52

0 556aa, >BBOV_IV006790... *

>Cluster 53

0 554aa, >BBOV_II005340... *

>Cluster 54

0 543aa, >BBOV_III004560... *

>Cluster 55

0 542aa, >BBOV_IV004970... *

>Cluster 56

0 539aa, >BBOV_IV000260... *

>Cluster 57

0 538aa, >BBOV_II003390... *

>Cluster 58

0 538aa, >BBOV_IV000230... *

>Cluster 59

0 537aa, >BBOV_III002510... *

>Cluster 60

0 534aa, >BBOV_IV006970... *

>Cluster 61

0 530aa, >BBOV_II003350... *

>Cluster 62

0 520aa, >BBOV_III009260... *

>Cluster 63

0 519aa, >BBOV_III004870... *

>Cluster 64

0 514aa, >BBOV_IV005830... *

>Cluster 65

0 508aa, >BBOV_IV005480... *

>Cluster 66

0 502aa, >BBOV_IV003870... *

>Cluster 67

0 498aa, >BBOV_III005850... *

>Cluster 68

0 495aa, >BBOV_IV006920... *

>Cluster 69

0 491aa, >BBOV_IV000400... *

>Cluster 70

0 485aa, >BBOV_III003170... *

>Cluster 71

0 485aa, >BBOV_IV010070... *

>Cluster 72

0 485aa, >BBOV_I003570... *

>Cluster 73

0 483aa, >BBOV_II006320... *

>Cluster 74

0 481aa, >BBOV_IV006780... *

>Cluster 75

0 480aa, >BBOV_III006800... *

>Cluster 76

0 478aa, >BBOV_II004460... *

>Cluster 77

0 476aa, >BBOV_II006920... *

>Cluster 78

0 468aa, >BBOV_III006620... *

>Cluster 79

0 453aa, >BBOV_IV004060... *

>Cluster 80

0 453aa, >BBOV_IV005840... *

>Cluster 81

0 447aa, >BBOV_III002820... *

>Cluster 82

0 445aa, >BBOV_II007650... *

>Cluster 83

0 443aa, >BBOV_IV008080... *

>Cluster 84

0 443aa, >BBOV_III011900... *

>Cluster 85

0 436aa, >BBOV_III007170... *

>Cluster 86

0 425aa, >BBOV_IV009940... *

>Cluster 87

0 423aa, >BBOV_II001020... *

>Cluster 88

0 423aa, >BBOV_IV001640... *

>Cluster 89

0 422aa, >BBOV_IV011970... *

>Cluster 90

0 421aa, >BBOV_IV008420... *

>Cluster 91

0 420aa, >BBOV_II002020... *

>Cluster 92

0 418aa, >BBOV_III003080... *

>Cluster 93

0 417aa, >BBOV_III004470... *

>Cluster 94

0 416aa, >BBOV_IV005040... *

>Cluster 95

0 413aa, >BBOV_III004540... *

>Cluster 96

0 412aa, >BBOV_II004560... *

>Cluster 97

0 411aa, >BBOV_III008640... *

>Cluster 98

0 409aa, >BBOV_III006110... *

>Cluster 99

0 406aa, >BBOV_I002450... *

>Cluster 100

0 402aa, >BBOV_III010250... *

>Cluster 101

0 399aa, >BBOV_II004930... *

>Cluster 102

0 398aa, >BBOV_III003780... *

>Cluster 103

0 395aa, >BBOV_II007380... *

>Cluster 104

0 395aa, >BBOV_III010470... *

>Cluster 105

0 390aa, >BBOV_I002930... *

>Cluster 106

0 385aa, >BBOV_IV005540... *

>Cluster 107

0 379aa, >BBOV_IV003010... *

>Cluster 108

0 377aa, >BBOV_III011860... *

>Cluster 109

0 374aa, >BBOV_III006320... *

>Cluster 110

0 369aa, >BBOV_IV009590... *

>Cluster 111

0 364aa, >BBOV_IV001810... *

>Cluster 112

0 349aa, >BBOV_IV003080... *

>Cluster 113

0 348aa, >BBOV_IV008190... *

>Cluster 114

0 341aa, >BBOV_IV003860... *

>Cluster 115

0 339aa, >BBOV_II007560... *

>Cluster 116

0 336aa, >BBOV_I002490... *

>Cluster 117

0 329aa, >BBOV_I001610... *

>Cluster 118

0 329aa, >BBOV_II005400... *

>Cluster 119

0 328aa, >BBOV_IV002290... *

>Cluster 120

0 323aa, >BBOV_III001060... *

>Cluster 121

0 321aa, >BBOV_III007940... *

>Cluster 122

0 320aa, >BBOV_I002820... *

>Cluster 123

0 314aa, >BBOV_III003950... *

>Cluster 124

0 312aa, >BBOV_IV004540... *

>Cluster 125

0 312aa, >BBOV_I000220... *

>Cluster 126

0 309aa, >BBOV_IV010530... *

>Cluster 127

0 308aa, >BBOV_III001540... *

>Cluster 128

0 306aa, >BBOV_III004840... *

>Cluster 129

0 301aa, >BBOV_II005860... *

>Cluster 130

0 297aa, >BBOV_II004540... *

>Cluster 131

0 293aa, >BBOV_I000910... *

>Cluster 132

0 292aa, >BBOV_IV000650... *

>Cluster 133

0 288aa, >BBOV_I005100... *

>Cluster 134

0 288aa, >BBOV_IV001650... *

>Cluster 135

0 281aa, >BBOV_IV001860... *

>Cluster 136

0 280aa, >BBOV_IV004160... *

>Cluster 137

0 276aa, >BBOV_IV003070... *

>Cluster 138

0 273aa, >BBOV_III003610... *

>Cluster 139

0 271aa, >BBOV_III005540... *

>Cluster 140

0 262aa, >BBOV_III002570... *

>Cluster 141

0 262aa, >BBOV_III010600... *

>Cluster 142

0 260aa, >BBOV_III004090... *

>Cluster 143

0 256aa, >BBOV_I000430... *

>Cluster 144

0 248aa, >BBOV_III007860... *

>Cluster 145

0 248aa, >BBOV_III009910... *

>Cluster 146

0 244aa, >BBOV_II001800... *

>Cluster 147

0 243aa, >BBOV_I004840... *

>Cluster 148

0 242aa, >BBOV_II002500... *

>Cluster 149

0 242aa, >BBOV_II000620... *

>Cluster 150

0 242aa, >BBOV_I004490... *

>Cluster 151

0 239aa, >BBOV_III008280... *

>Cluster 152

0 236aa, >BBOV_IV003270... *

>Cluster 153

0 234aa, >BBOV_IV011510... *

>Cluster 154

0 230aa, >BBOV_III004660... *

>Cluster 155

0 225aa, >BBOV_II001250... *

>Cluster 156

0 225aa, >BBOV_II004070... *

>Cluster 157

0 223aa, >BBOV_IV000600... *

>Cluster 158

0 221aa, >BBOV_V000090... *

>Cluster 159

0 217aa, >BBOV_IV010550... *

>Cluster 160

0 213aa, >BBOV_II005810... *

>Cluster 161

0 213aa, >BBOV_III005750... *

>Cluster 162

0 212aa, >BBOV_I001770... *

>Cluster 163

0 211aa, >BBOV_III001740... *

>Cluster 164

0 207aa, >BBOV_III004170... *

>Cluster 165

0 207aa, >BBOV_II006260... *

>Cluster 166

0 196aa, >BBOV_IV000530... *

>Cluster 167

0 195aa, >BBOV_I005030... *

>Cluster 168

0 193aa, >BBOV_I000240... *

>Cluster 169

0 190aa, >BBOV_I002860... *

>Cluster 170

0 188aa, >BBOV_II004080... *

>Cluster 171

0 187aa, >BBOV_III007190... *

>Cluster 172

0 186aa, >BBOV_IV011290... *

>Cluster 173

0 180aa, >BBOV_IV004220... *

>Cluster 174

0 179aa, >BBOV_III002560... *

>Cluster 175

0 176aa, >BBOV_IV002410... *

>Cluster 176

0 175aa, >BBOV_III006650... *

>Cluster 177

0 169aa, >BBOV_III010670... *

>Cluster 178

0 167aa, >BBOV_IV000800... *

>Cluster 179

0 164aa, >BBOV_III009860... *

>Cluster 180

0 163aa, >BBOV_I001760... *

>Cluster 181

0 162aa, >BBOV_I005050... *

>Cluster 182

0 159aa, >BBOV_IV008910... *

>Cluster 183

0 152aa, >BBOV_IV004930... *

>Cluster 184

0 151aa, >BBOV_IV006660... *

>Cluster 185

0 149aa, >BBOV_I004080... *

>Cluster 186

0 146aa, >BBOV_III010680... *

>Cluster 187

0 145aa, >BBOV_IV005100... *

>Cluster 188

0 145aa, >BBOV_I001340... *

>Cluster 189

0 141aa, >BBOV_II005000... *

>Cluster 190

0 140aa, >BBOV_III000180... *

>Cluster 191

0 135aa, >BBOV_II003270... *

>Cluster 192

0 134aa, >BBOV_III006250... *

>Cluster 193

0 132aa, >BBOV_III001350... *

>Cluster 194

0 129aa, >BBOV_V000300... *

>Cluster 195

0 129aa, >BBOV_IV004320... *

>Cluster 196

0 128aa, >BBOV_IV002190... *

>Cluster 197

0 127aa, >BBOV_IV002860... *

>Cluster 198

0 125aa, >BBOV_IV007200... *

>Cluster 199

0 120aa, >BBOV_II007490... *

>Cluster 200

0 118aa, >BBOV_III007130... *

>Cluster 201

0 113aa, >BBOV_III009270... *

>Cluster 202

0 112aa, >BBOV_IV004410... *

>Cluster 203

0 111aa, >BBOV_III007490... *

>Cluster 204

0 110aa, >BBOV_I004740... *

>Cluster 205

0 110aa, >BBOV_I002260... *

>Cluster 206

0 94aa, >BBOV_IV002350... *

>Cluster 207

0 90aa, >BBOV_IV003900... *

>Cluster 208

0 77aa, >BBOV_II003780... *

>Cluster 209

0 76aa, >BBOV_III002410... *

>Cluster 210

0 75aa, >BBOV_I002150... *

>Cluster 211

0 70aa, >BBOV_III000120... *

>Cluster 212

0 59aa, >BBOV_I001590... *

>Cluster 213

0 51aa, >BBOV_II004750... *
